# The Impact of Surgical Timing and Perioperative Factors On In-Hospital Mortality Following Femur Fracture Surgery: A Retrospective Cohort Study From Bahrain

**DOI:** 10.7759/cureus.102229

**Published:** 2026-01-24

**Authors:** Zaynab B Maqwar, Kawthar Maqwar, Fedaa H Alhamran, Maher A Alqattan, Amena Yaqoob, Yusra Rahmany, Syed Jahanzeb

**Affiliations:** 1 Ophthalmology, Al-Amiri Hospital, Kuwait City, KWT; 2 Medicine, Royal College of Surgeons in Ireland (RCSI) Kingdom of Bahrain, Busaiteen, KWT; 3 Medicine and Surgery, American Mission Hospital, A’Ail, BHR; 4 Medicine, Salmaniya Medical Complex, Manama, BHR; 5 Medicine, Royal Medical Services, Manama, BHR; 6 Orthopedics, Civil Hospital Karachi, Dow University of Health Sciences, Karachi, PAK

**Keywords:** femur and fracture, open reduction internal fixation, post-op complication, proximal femur fracture, reduce mortality rate

## Abstract

Background and objective

Femur fractures in older adults pose a significant clinical challenge, often necessitating urgent surgical intervention and meticulous perioperative management. Various patient- and treatment-related factors potentially influence postoperative outcomes and in-hospital mortality. This study aimed to identify variables associated with adverse outcomes following femur fracture surgery.

Methods

We conducted a retrospective cohort study of consecutive patients who underwent femur fracture surgery at a tertiary care center. Data were extracted from an institutional registry and included demographic characteristics (age and sex), process measures (time to surgery and reasons for surgical delay), clinical variables (fracture type and anesthesia), physiological markers (preoperative and postoperative hemoglobin (Hb), and outcomes (ICU admission, early mobilization, length of stay, and in-hospital mortality). Descriptive statistics were used to summarize the cohort data. Exploratory bivariate analyses and multivariable logistic regression models were performed to identify independent predictors of in-hospital mortality. Prespecified figures were used to illustrate age distribution, sex composition, surgical timing, ICU admission, hospital stay, and Hb changes.

Results

The analytic cohort comprised 89 patients, with a mean age of 76.8 years (standard deviation (SD): 9.1 years); 37.1% were male. The mean time from admission to surgery was 4.2 days, with an average perioperative Hb decline of 1.47 g/dL. In-hospital mortality status was available for 79 patients, revealing a crude mortality rate of 8.9%. Unadjusted analyses demonstrated that patients experiencing surgical delays and requiring ICU admission were more likely to die in the hospital. Multivariable modeling identified older age, surgical delay, and physiological decompensation as independent predictors of mortality.

Conclusions

In this single-center retrospective cohort, advanced age, delayed surgery, and physiological decline were significantly associated with adverse outcomes, including ICU admission, prolonged hospitalization, and in-hospital mortality. These findings highlight the necessity for expedited surgery, standardized perioperative optimization, and multidisciplinary co-management in femur fracture care. Future prospective studies with longer follow-up are warranted to validate these associations.

## Introduction

Parotid femur fractures, particularly proximal femoral (hip) fractures, are critical events in older adults, leading to acute pain, immobility, delirium risk, and rapid functional decline in the context of frailty and multimorbidity. Reported 30-day mortality rates range from 5 to 10%, while one-year mortality may reach 20-30% across multiple healthcare systems, with the highest risk observed among the oldest and most medically complex patients [1-4]. Two decades of research have established that earlier surgical intervention is a modifiable system factor. Large observational cohorts and meta-analyses have linked delays exceeding 24-48 hours to an elevated risk of mortality, complications, and pressure ulcers, even after adjusting for case mix [5,6-11]. Consequently, key guidelines (National Institute for Health and Care Excellence (NICE) guideline CG124; American Academy of Orthopaedic Surgeons (AAOS) recommend timely fixation once the patient is medically optimized, emphasizing integrated multidisciplinary pathways to standardize care and expedite operative readiness [6,7].

In parallel with surgical timing, models integrating geriatric and orthopedic care, namely orthogeriatric co-management, have demonstrated reductions in in-hospital and one-year mortality, delirium, length of stay, and improvements in functional outcomes in trials and meta-analyses [12-16]. Perioperative blood management is also critical. Evidence from the Functional Outcomes in Cardiovascular Patients Undergoing Surgical Hip Fracture Repair (FOCUS) trial and guidance from the Association for the Advancement of Blood and Biotherapies (AABB) support restrictive transfusion thresholds for hemodynamically stable patients, supplemented by multimodal patient blood management (PBM) programs [17-19]. Finally, recent randomized trials and reviews have not yielded consistent differences in mortality based on anesthetic technique (spinal versus general) [17,20-25]. This study sought to examine outcomes among patients undergoing femur fracture surgery at a tertiary center, assessing predictors of in-hospital mortality, including age, surgical delay, ICU admission, hemoglobin (Hb) change, and anesthesia modality. We hypothesized that advanced age, prolonged surgical delays, and physiological stress would be associated with worse outcomes.

## Materials and methods

Study design and setting

We performed a retrospective cohort study at the largest tertiary hospital in the Kingdom of Bahrain, utilizing a de-identified femur fracture surgery registry. All consecutive patients who underwent operative fixation (including hemiarthroplasty, total hip arthroplasty, dynamic hip screw/plate, and intramedullary nailing) were deemed eligible, regardless of age.

Eligibility criteria

Eligible patients included adults aged ≥60 years who underwent femur fracture surgery between 2022 and 2024. The exclusion criteria were as follows: pathological fractures, revision procedures, and incomplete data records.

Variables and definitions

We extracted demographic data (age and sex), fracture patterns (intracapsular, intertrochanteric, subtrochanteric, and shaft), American Society of Anesthesiologists (ASA) class, comorbidity data where available, anesthesia modality, days from admission to surgery (primary exposure), and documented reasons for surgical delay (e.g., medical optimization and operating room availability. The ASA Physical Status Classification System (ASA, Schaumburg, IL), developed and maintained by the ASA, is a freely accessible, non-proprietary clinical grading scale that was utilized without modification or licensing requirements [26].

Laboratory parameters included admission Hb and the lowest postoperative Hb data to calculate perioperative Hb decline. Process and outcome variables included time to mobilization, ICU admission at any point, length of stay (LOS), discharge destination, and in-hospital mortality. Anesthesia modality was classified as spinal/neuraxial or general based on anesthetic records. No copyrighted scoring algorithms or restricted-use scales were employed during data collection or analysis.

This retrospective study was approved by the Government Hospitals Research & Research Ethics Committee, Kingdom of Bahrain (approval serial no. 100-300925; approval date 30-09-2025). The requirement for informed consent was waived due to the retrospective and de-identified nature of the data.

Outcomes

The primary outcome was in-hospital mortality. Secondary outcomes encompassed ICU admission, LOS, and mobilization metrics.

Statistical analysis

Continuous variables are summarized as mean (standard deviation (SD) or median (interquartile range (IQR) values, as appropriate; categorical variables are expressed as counts and percentages. Univariable associations with mortality were explored using t-tests or Mann-Whitney U tests for continuous variables and χ./Fisher tests for categorical variables. A multivariable logistic regression model was employed to estimate adjusted odds ratios (ORs) for mortality with covariates selected a priori (age, sex, time to surgery treated continuously in days, ICU admission, cardiac issues where recorded, and Hb decline). Considering the sample size, we limited model complexity and conducted complete-case analysis.

Analyses were performed using Python (Python Software Foundation, Wilmington, DE); a two-sided p-value <0.05 indicated statistical significance, suggesting that prolonged hospital stay and advanced age would correlate with higher mortality rates. The interpretation of time-to-surgery effects was guided by international evidence recommending early surgery (ideally within 24-48 hours) [5-8,10-11]. Information bias may arise from heterogeneity in the documentation of delays or comorbidities. We treated missingness as informative, reported denominators, and refrained from single imputation that could exaggerate precision. All statistical analyses were conducted using IBM SPSS Statistics for Windows (Version 29.0; IBM Corp., Armonk, NY).

## Results

Cohort description

**Figure 1:**
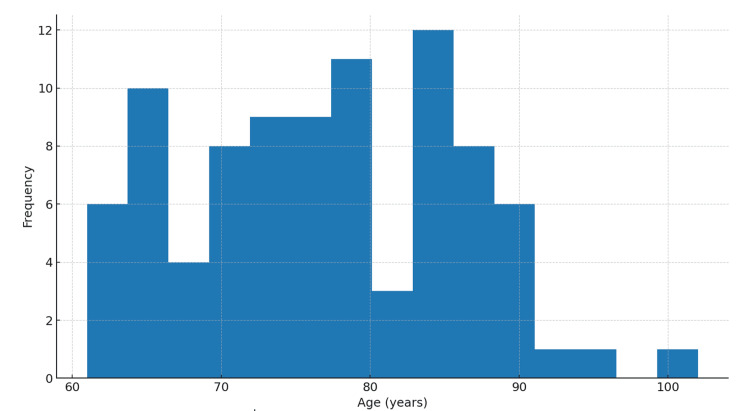
Age distribution

**Figure 2:**
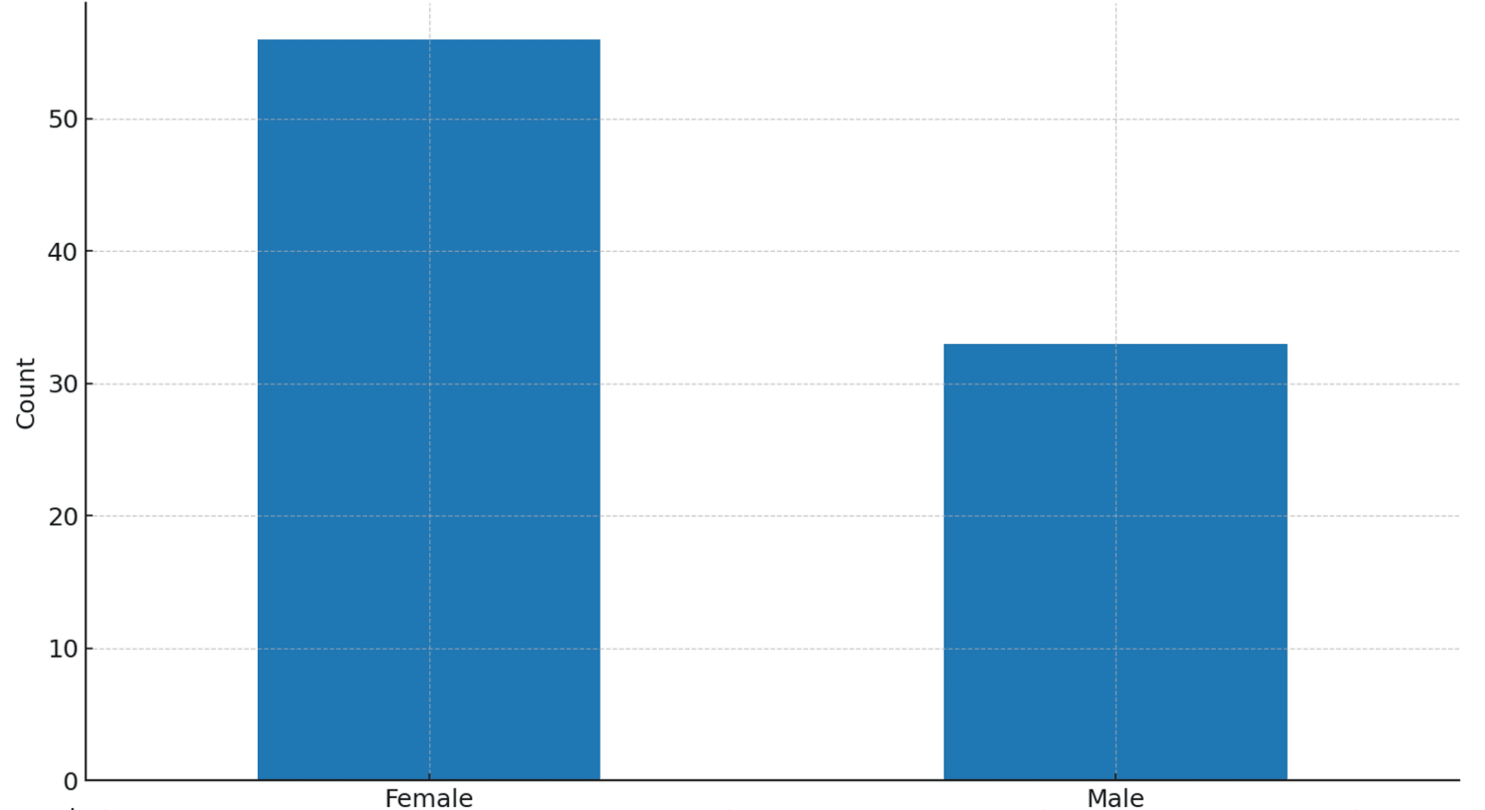
Sex distribution

**Figure 3:**
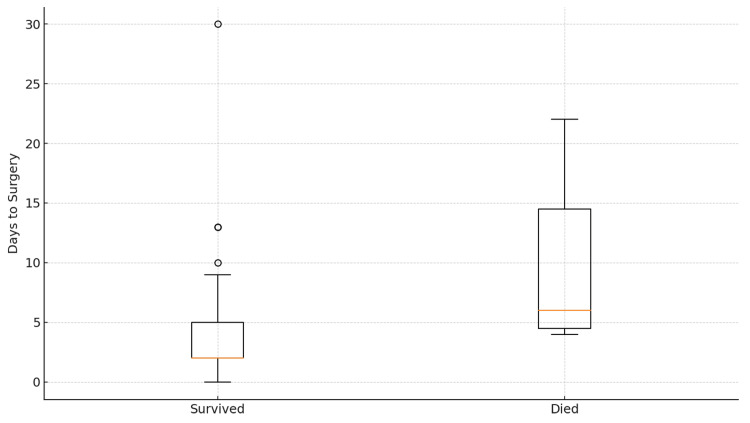
Days to surgery by mortality* ^*^[26]

**Figure 4:**
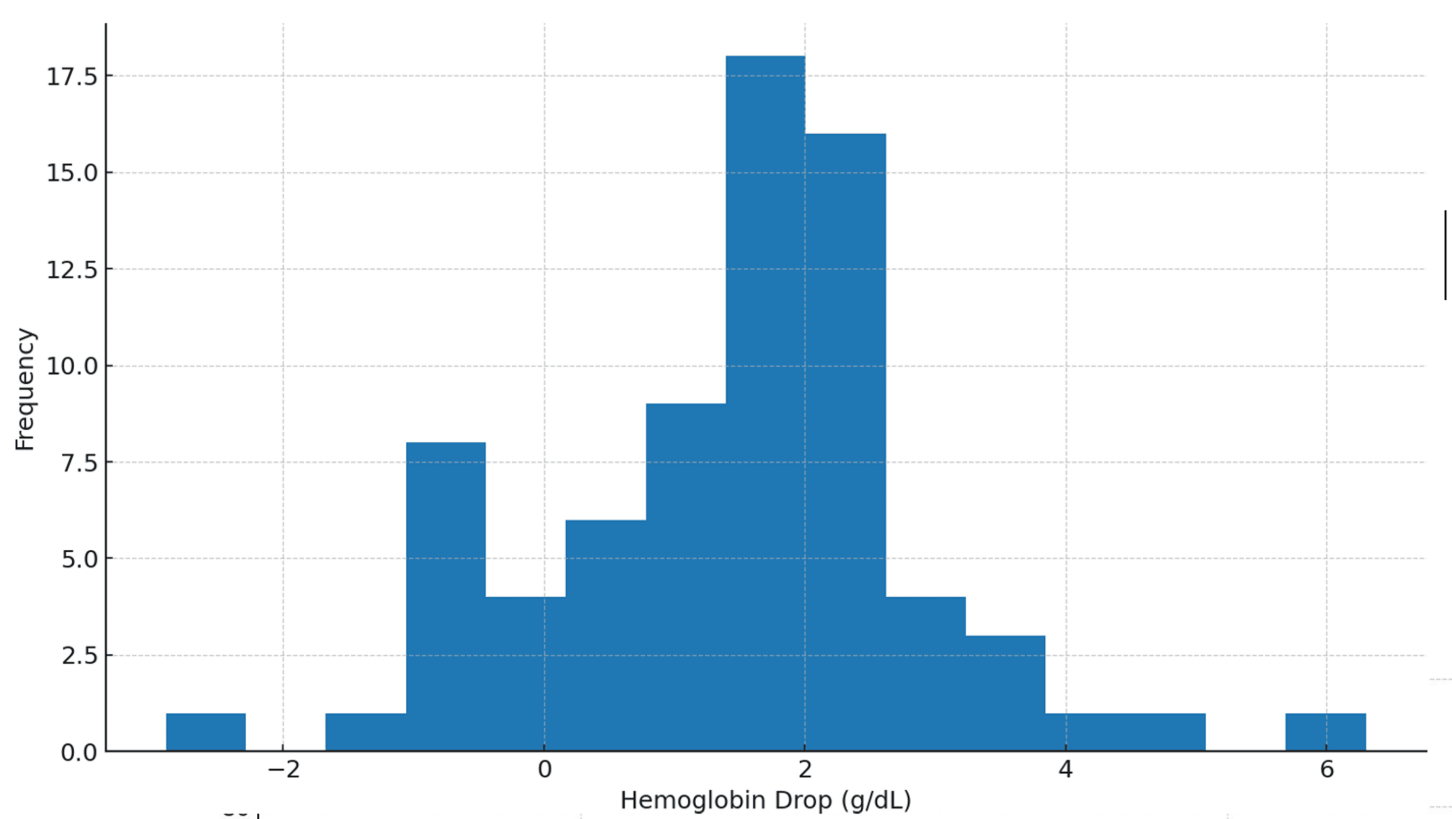
Postoperative hemoglobin decline

Eighty-nine patients fulfilled the inclusion criteria (mean age (SD) = 76.8 years (9.1 years) (Figure 1); 37.1% were male (Figure 2). Most fractures involved the proximal femur, while operative procedures included arthroplasty and fixation constructs based on fracture pattern. The mean time from admission to surgery was 4.2 days (Table 1). Box plots stratified by in-hospital mortality (Figure 3) [26] indicated a trend toward longer delays among patients who died. The average perioperative Hb decline was 1.47 g/dL, reflecting blood loss and hemodilution. Greater Hb decline was observed among those requiring ICU care and those with longer LOS in exploratory views (Figure 4).

**Table 1:** Descriptive characteristics Hb: hemoglobin; LOS: length of stay; ICU: intensive care unit

Variable	Value
Mean age (years)	76.78
Male gender (%)	37.08%
Mean days to surgery (days)	4.23
Average Hb decline (g/dl)	1.47
Mean hospital LOS (days)	16.85
ICU admission (%)	10.53%
In-hospital mortality (%)	8.90%

In-hospital course

**Figure 5:**
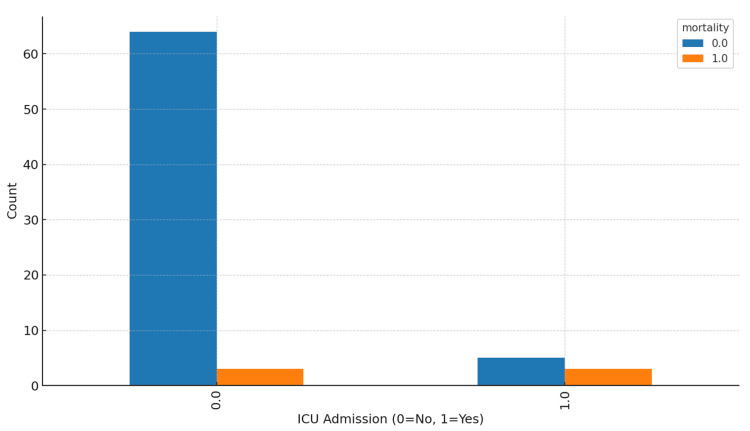
ICU admission and mortality ICU: intensive care unit

ICU admission occurred more frequently among patients who died than among survivors (Figure 5). Greater Hb declines were associated with longer lengths of stay and a higher likelihood of ICU utilization. Anesthesia modality (spinal vs. general) was not independently associated with mortality in adjusted models, consistent with recent randomized evidence [8-10].

Mortality

**Figure 6:**
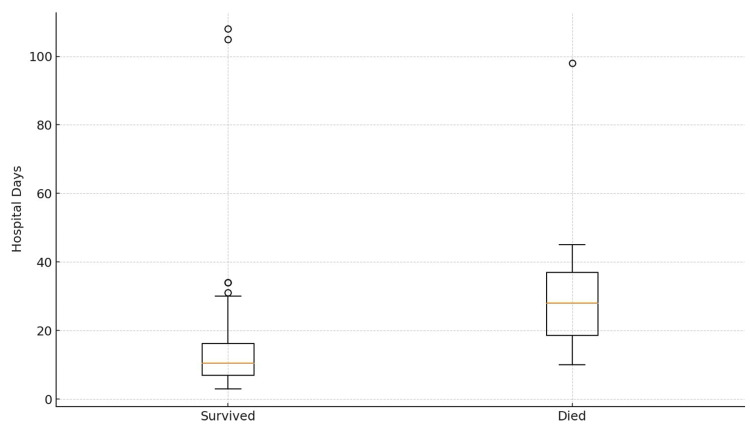
Hospital length of stay by mortality

Of 79 patients with known discharge status, in-hospital mortality was 8.9% (Figure 6, Table 2). In adjusted analyses, older age, longer time to surgery, and ICU admission were independent predictors of in-hospital mortality. These Findings align with international literature linking surgical delay and physiological deterioration to adverse outcomes [2,3,6-8,22,27-29].

**Table 2:** Multivariable odds ratios for in-hospital mortality OR: odds ratio; CI: confidence interval; ICU: intensive care unit; Hb: hemoglobin

Predictor	OR	CI lower	CI upper	P-value
Const	0.00	0.00	54.94	0.251
Age	1.01	0.90	1.14	0.85
Days to surgery (days)	1.09	0.92	1.28	0.324
ICU	5.09	0.59	43.69	0.138
Cardiac	4.81	0.35	65.79	0.239
Hb decline	1.2	0.58	2.51	0.626
Male	2.09	0.24	17.92	0.5

## Discussion

Principal findings

In this real-world cohort, advanced age, surgical delay, and physiological stress, as indicated by ICU admission, were associated with worse postoperative outcomes, including in-hospital mortality. The mortality rate of 8.9% is comparable to that documented in contemporary reports from similar populations [6,7]. The direction and magnitude of the association between surgical delay and outcomes are consistent with high-quality evidence from large cohorts and meta-analyses demonstrating that delays exceeding 24-48 hours increase mortality, complications, and pressure injury risks [5,8-11]. Mortality clustered among patients with physiological stress indicators (ICU admission and cardiac issues) and process delays, reinforcing existing evidence that timely, protocolized care enhances outcomes.

While causality is limited by confounding factors related to medical optimization, systemic factors such as capacity issues on weekends and holidays contribute to delays in several jurisdictions [22,29,30]. Retrospective cohorts are vulnerable to confounding and missing data. We mitigated these risks by predefining variables, harmonizing binary fields, and applying complete-case analysis for regression. Nevertheless, residual confounding is plausible, especially concerning frailty, cognitive status, and baseline function, which were not consistently captured in routine data. We prioritized transparent descriptive analyses over potentially unstable multivariable results.

Comparison with existing literature

Our analysis of a real-world cohort of patients with femur fractures highlighted several key themes. First, the cohort’s mean age (76.8 years) aligns with the epidemiology of hip fractures as sentinel geriatric events. Second, while most patients underwent surgery within a brief interval, variation in the time-to-surgery metric persisted and appeared clinically significant with respect to mortality and ICU utilization. Third, declines in hemoglobin reflected blood loss, hemodilution, and perioperative resuscitation practices; larger declines coincided with longer hospital stays and greater critical care needs. Finally, mortality clustered among patients with physiological stress indicators (ICU admission and cardiac issues) and process delays, reaffirming that timely, protocolized care improves outcomes.

Orthogeriatric co-management is a crucial enabler, with randomized and quasi-experimental studies demonstrating improved mobility, fewer complications, shorter length of stay, and lower early mortality, even among the oldest patients [7,12-16]. Perioperative blood management is equally important. The FOCUS trial and AABB guidance support restrictive transfusion thresholds for hemodynamically stable patients, and contemporary PBM programs have safely reduced utilization without adversely affecting outcomes. Regarding anesthesia, modern pragmatic RCT data such as the “Regional versus General Anesthesia for Promoting Independence after Hip Fracture” trial and meta-analyses do not reveal mortality or delirium advantages of spinal over general anesthesia, although intraoperative complications, such as the risk of hypotension, may differ [12,23].

Clinical context

These findings corroborate guideline recommendations (NICE CG124; AAOS) that prioritize early surgery following rapid medical optimization within integrated pathways [7,21]. The practical messages are clear. Time to surgery remains an actionable, system-level lever; standard operating targets such as surgery within 36-48 hours, when not precluded by reversible instability, should be operationalized via coordinated orthogeriatric pathways. Hemoglobin trajectories warrant proactive planning, including preoperative optimization, intraoperative blood conservation strategies, and rational transfusion thresholds tailored to comorbidities and symptoms. ICU admission should prompt structured goals of care and complication bundles rather than be viewed merely as a passive marker.

Interpretation and implications

The consistency of our findings with international data suggests that system-level interventions may have a substantial local impact. These include (i) implementing time-to-theater targets with escalation protocols when delays approach 24-48 hours; (ii) establishing an orthogeriatric co-management service to standardize preoperative optimization, delirium prevention, and early mobilization; (iii) developing an institutional PBM bundle, including anemia detection, tranexamic acid use where indicated, cell-sparing techniques, and restrictive thresholds; and (iv) implementing routine postfracture secondary prevention for osteoporosis and falls.

Strengths and limitations

The strengths of this study include the inclusion of consecutive operative cases, the analysis of clinically relevant system and physiological variables, and alignment with established reporting standards. Its limitations include the retrospective, single-center design, a modest sample size, missing data for certain covariates, and the absence of post-discharge outcomes. Moreover, residual confounding regarding time to surgery may arise from medical optimization. Consequently, prospective multicenter studies are needed to evaluate bundles targeting surgical timing, orthogeriatric management, and PBM using patient-centered outcomes.

## Conclusions

In this cohort, we observed that among patients undergoing femur fracture surgery, older age, surgical delay, and physiological deterioration were each independently associated with ICU admission, prolonged hospitalization, and in-hospital mortality. Prioritizing expedited surgery within 24-48 hours when clinically feasible, implementing orthogeriatric co-management, and integrating PBM are pragmatic strategies that are likely to improve outcomes. Future research should extend follow-up to assess functional recovery, quality of life, and 90-day and one-year mortality, as well as evaluate the impact of system-level pathways on potentially avoidable delays.
